# Supplementary material for: Ground-truth encoding of self-motion in the primate cerebellar nodulus and uvula
Source: Nat Commun. 2026 Feb 25;17:3166. doi: 10.1038/s41467-026-69909-9 (PMC13046849; doi:10.1038/s41467-026-69909-9)
Supplement: Supplementary file 1 — Supplementary Information [file 41467_2026_69909_MOESM1_ESM.pdf]

Ground-truth encoding of self-motion in the primate cerebellar nodulus and uvula

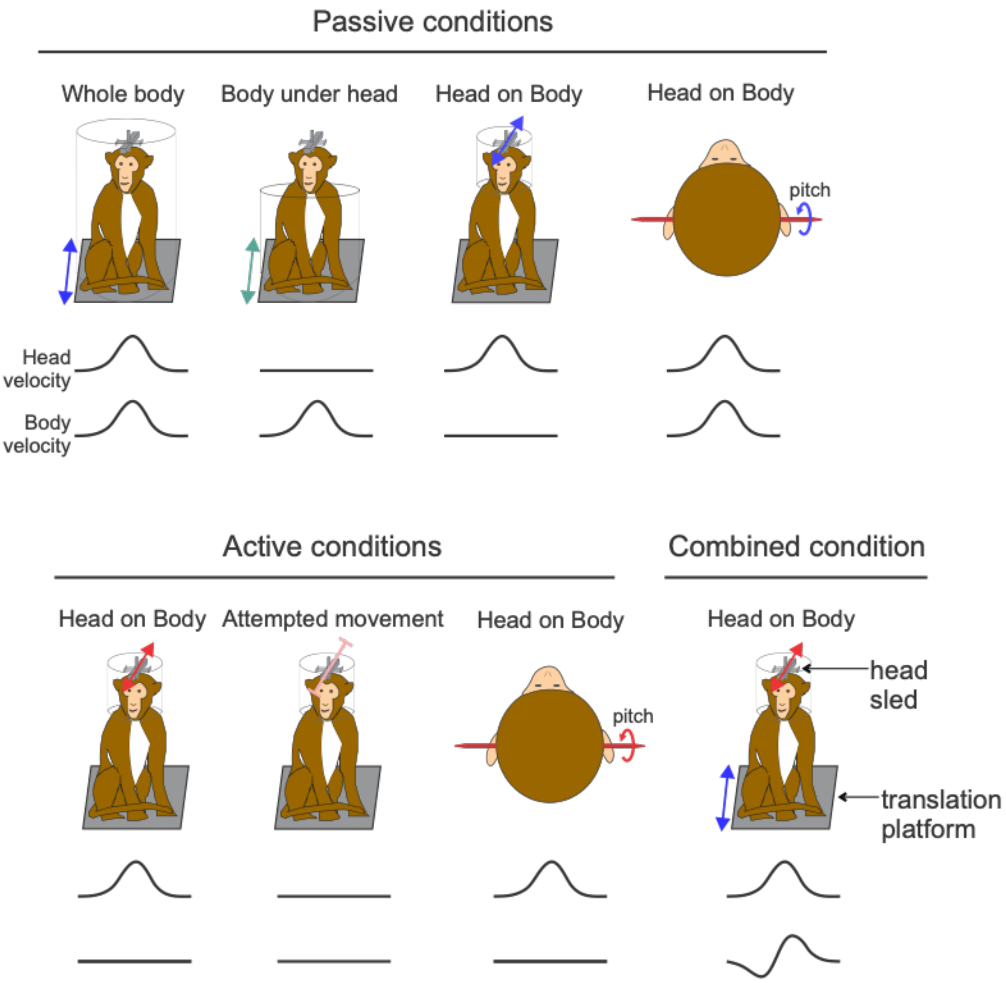

Supplementary Fig. 1. Illustration of the experimental setup to apply passive vestibular and proprioceptive stimulation, and allow the monkey to perform active head movements Experimental approaches to apply vestibular (whole body), proprioceptive (body under head), and combined (head on body) translations, as well as head on body pitch. Integration of motor command information was tested by allowing the monkey to make, or attempt to make, active head on body movements alone or simultaneously paired with passive vestibular stimulation.

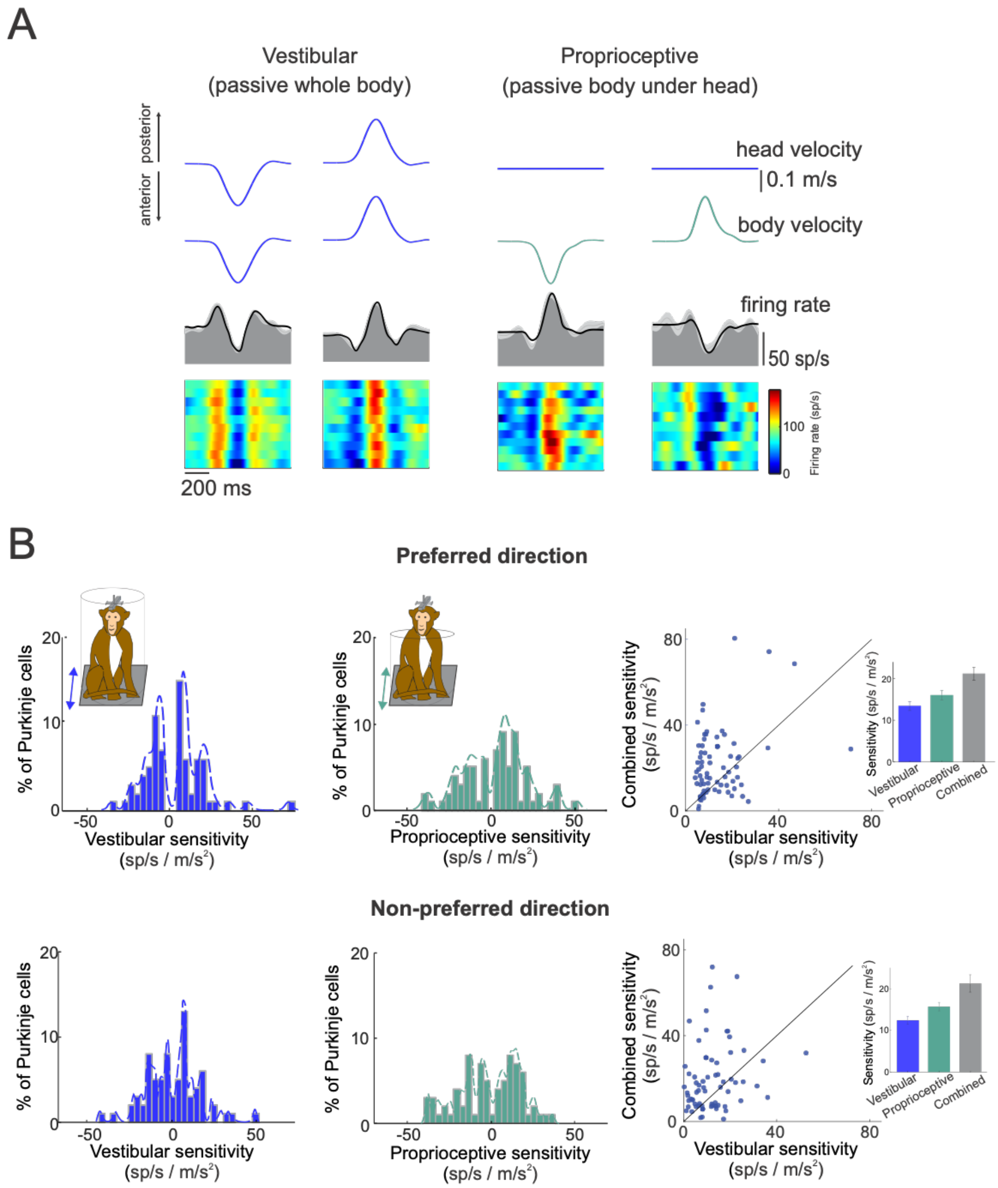

Supplementary Fig. 2. Responses of Purkinje cell simple spikes to vestibular and neck proprioceptive stimulation in the anteroposterior direction

(A) Response of an example Purkinje cell to vestibular and proprioceptive stimulation. Head and body translation velocity are shown in the top two rows, and simple spike firing rate (gray shaded area) along with the linear estimation of the firing rate based on head motion (superimposed black trace), is shown in the bottom row. Heat maps illustrate simple spike firing rates for each motion trial. **(B)** Distribution of Purkinje cell responses to passive vestibular and proprioceptive stimulation during anteroposterior motion in both the preferred and non-preferred directions, and scatter plots demonstrating responses to combined vestibular and proprioceptive stimulation (head on body motion) are generally enhanced (above the unity line) relative to vestibular stimulation alone. Bar graph inset compares sensitivities to vestibular stimulation alone, proprioceptive stimulation along, and combined stimulation.

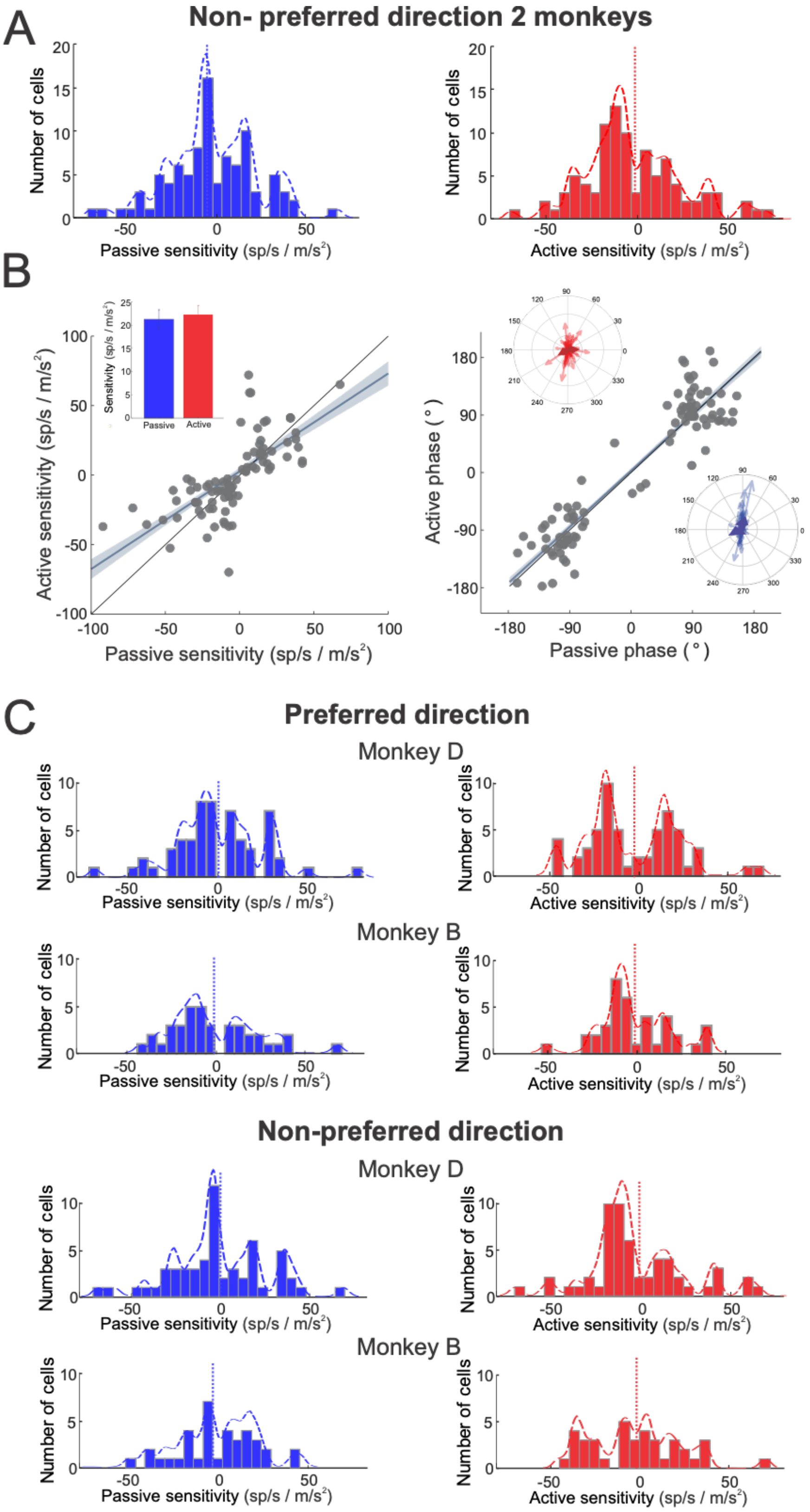
A
Non- preferred direction 2 monkeys
Number of cells
Passive sensitivity (sp/s / m/s²)
Active sensitivity (sp/s / m/s²)
B
Active sensitivity (sp/s / m/s²)
Passive sensitivity (sp/s / m/s²)
Sensitivity (sp/s / m/s²)
Passive
Active
Active phase (°)
Passive phase (°)
C
Preferred direction
Monkey D
Monkey B
Non-preferred direction
Monkey D
Monkey B
Number of cells
Passive sensitivity (sp/s / m/s²)
Active sensitivity (sp/s / m/s²)

Supplementary Fig. 3. Purkinje cell responses to active and passive translations in the non-preferred movement direction and responses for each monkey individually

(A) Distribution of sensitivities across active and passive head on body motion conditions in the non-preferred direction for both monkeys combined. **(B)** Passive vs. active Purkinje cell sensitivities and phases to head motion in the non-preferred movement direction. Gray line and shading represent the linear fit ±95% confidence interval. Insets: Bar graph demonstrates mean passive vs. active sensitivities, and polar plots show the magnitude (vector length) and the phase (angle) of Purkinje cell responses to passive and active head motion in the non-preferred movement direction. **(C)** Distributions of sensitivities for active and passive head on body motion in both the preferred and non-preferred directions for each monkey individually (Monkey D and Monkey B).

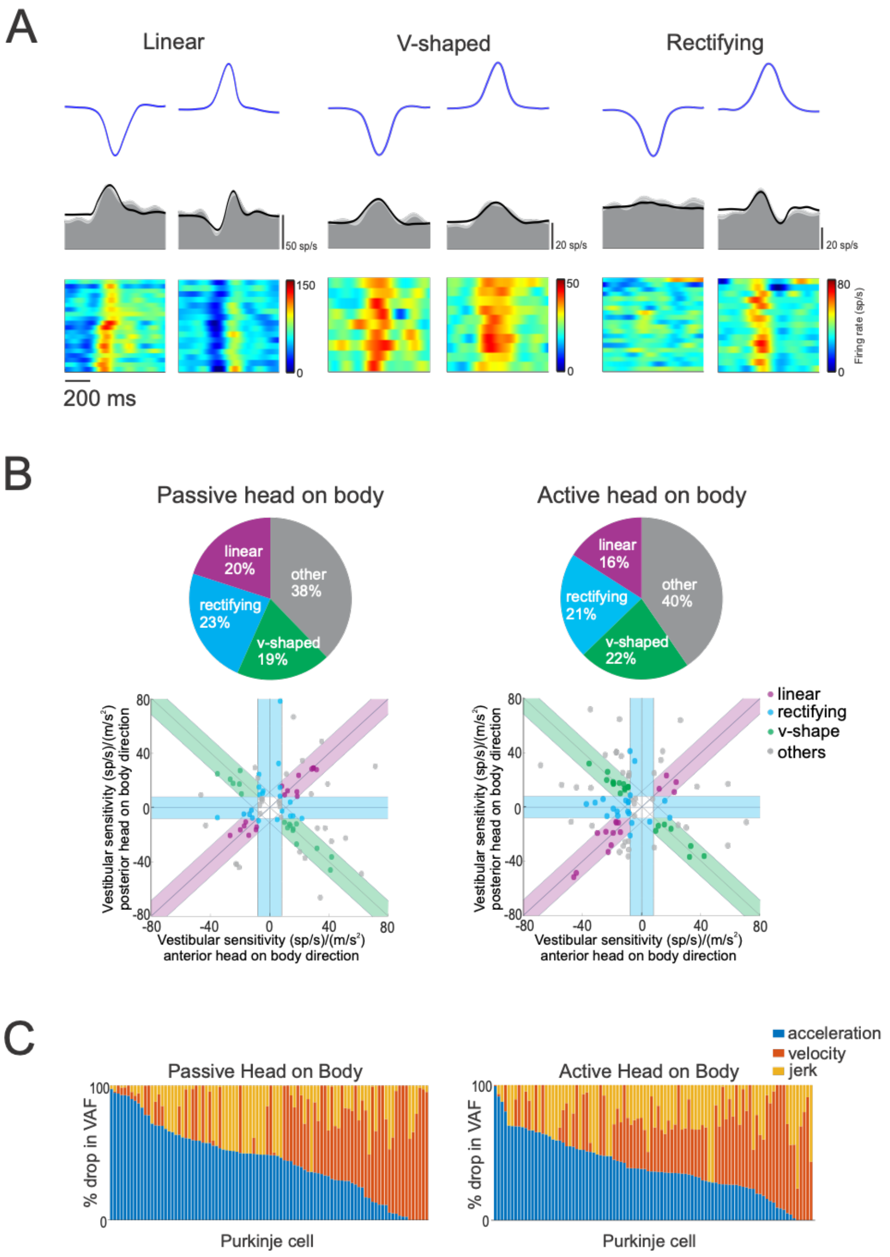

Supplementary Fig. 4. Heterogeneity of responses of Purkinje cell simple spikes to passive and active head on body motion (**A**) Three example neurons that show linear, v-shaped, and rectifying responses to head on body motion. Head and body translation velocity are shown in the top two rows, and simple spike firing rate (gray shaded area) along with the linear estimation of the firing rate based on head motion (superimposed black trace), is shown in the bottom row. Heat maps illustrate simple spike firing rates for each motion trial. **(B)** Classification of cells into linear, rectifying, v-shaped, and other based on sensitivities to passive and active head on body motion. Pie charts show proportions of cells classified into each category. (**C**) The decrease in variance accounted for (VAF) when each kinematic term (acceleration, velocity, and jerk) was systematically removed from the linear regression model for passive and active head on body motion.

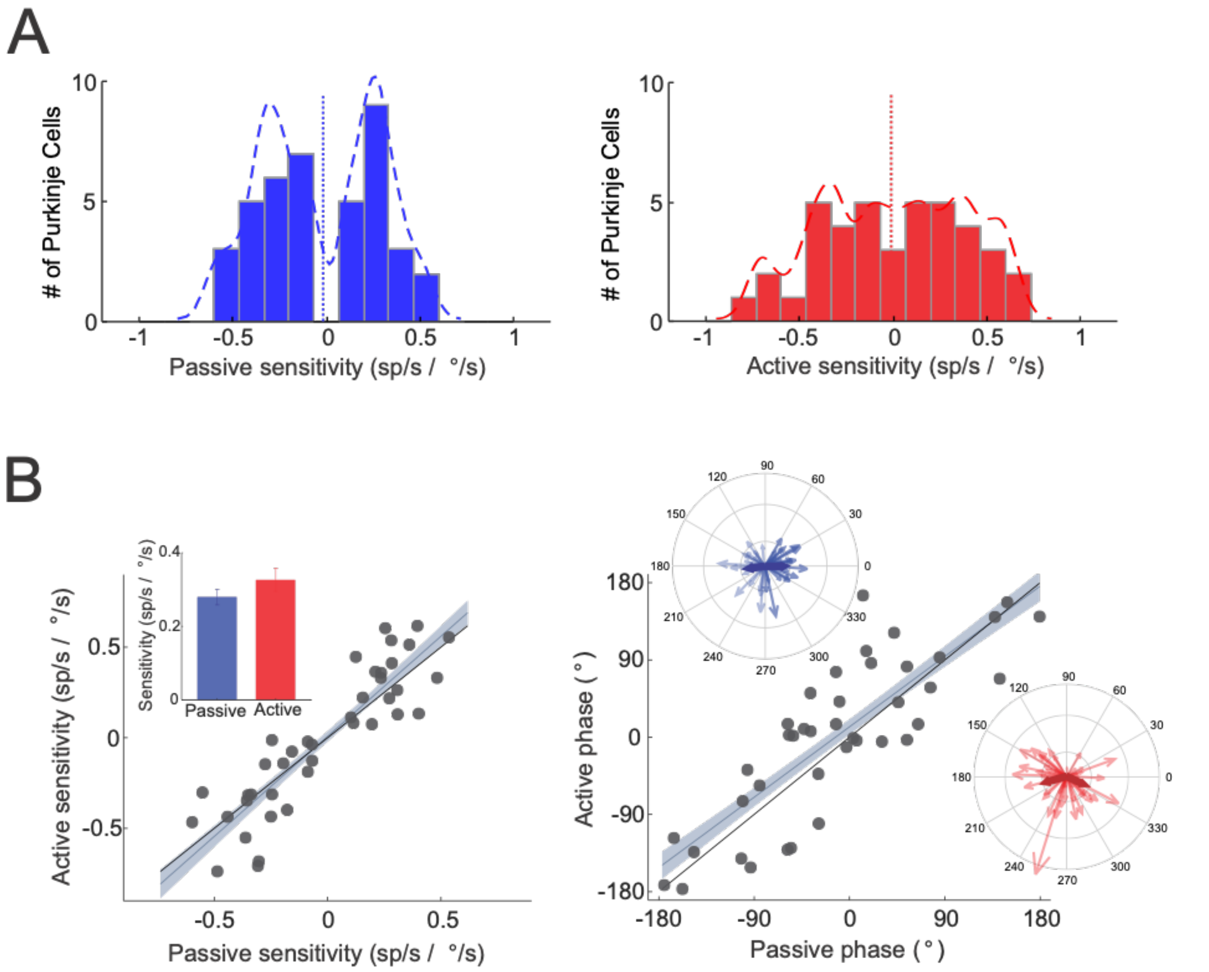

Supplementary Fig. 5. Purkinje cell responses to passive and active dynamic head pitch in the non-preferred moment direction

(A) Distribution of firing rates across passive and active head pitch conditions**. (B)** Passive vs. active Purkinje cell sensitivities and phases to head pitch in the non-preferred movement direction. Gray line and shading represent the linear fit ±95% confidence interval. Insets: Bar graph demonstrates mean ± standard error passive vs. active sensitivities, and polar plots show the magnitude (vector length) and the phase (angle) of Purkinje cell responses to passive (blue) and active (red) head motion in the non-preferred movement direction.
