# Supplementary material for: Ground-truth encoding of self-motion in the primate cerebellar nodulus and uvula
Source: Nat Commun. 2026 Feb 25;17:3166. doi: 10.1038/s41467-026-69909-9 (PMC13046849; doi:10.1038/s41467-026-69909-9)
Supplement: Supplementary file 2 — Reporting Summary [file 41467_2026_69909_MOESM2_ESM.pdf]

Corresponding author(s): Kathleen Cullen

Last updated by author(s): 01/21/2026

Reporting Summary

Nature Portfolio wishes to improve the reproducibility of the work that we publish. This form provides structure for consistency and transparency in reporting. For further information on Nature Portfolio policies, see our Editorial Policies and the Editorial Policy Checklist.

Statistics

For all statistical analyses, confirm that the following items are present in the figure legend, table legend, main text, or Methods section.

n/a	Confirmed	
☐	☒	The exact sample size (*n*) for each experimental group/condition, given as a discrete number and unit of measurement
☐	☒	A statement on whether measurements were taken from distinct samples or whether the same sample was measured repeatedly
☐	☒	The statistical test(s) used AND whether they are one- or two-sided<br>*Only common tests should be described solely by name; describe more complex techniques in the Methods section.*
☒	☐	A description of all covariates tested
☐	☒	A description of any assumptions or corrections, such as tests of normality and adjustment for multiple comparisons
☐	☒	A full description of the statistical parameters including central tendency (e.g. means) or other basic estimates (e.g. regression coefficient) AND variation (e.g. standard deviation) or associated estimates of uncertainty (e.g. confidence intervals)
☐	☒	For null hypothesis testing, the test statistic (e.g. *F*, *t*, *r*) with confidence intervals, effect sizes, degrees of freedom and *P* value noted<br>*Give P values as exact values whenever suitable.*
☒	☐	For Bayesian analysis, information on the choice of priors and Markov chain Monte Carlo settings
☒	☐	For hierarchical and complex designs, identification of the appropriate level for tests and full reporting of outcomes
☐	☒	Estimates of effect sizes (e.g. Cohen's *d*, Pearson's *r*), indicating how they were calculated

Our web collection on statistics for biologists contains articles on many of the points above.

Software and code

Policy information about availability of computer code

Data collection	Single unit neural activity were recorded (Intan RHS system, v3.4.0) from the brain in rhesus monkeys during head and body movement.
Data analysis	After recordings, off-line analyses of data were performed using custom algorithms (MATLAB v9.8, MathWork). The data are deposited in the Figshare database and codes in GitHub.

For manuscripts utilizing custom algorithms or software that are central to the research but not yet described in published literature, software must be made available to editors and reviewers. We strongly encourage code deposition in a community repository (e.g. GitHub). See the Nature Portfolio guidelines for submitting code & software for further information.

Data

Policy information about availability of data

All manuscripts must include a data availability statement. This statement should provide the following information, where applicable:

- Accession codes, unique identifiers, or web links for publicly available datasets
- A description of any restrictions on data availability
- For clinical datasets or third party data, please ensure that the statement adheres to our policy

The processed data that support the findings of this study are available in figshare with the identifier https://doi.org/10.6084/m9.figshare.30811046

Research involving human participants, their data, or biological material

Policy information about studies with human participants or human data. See also policy information about sex, gender (identity/presentation), and sexual orientation and race, ethnicity and racism.

Reporting on sex and gender	N/A
Reporting on race, ethnicity, or other socially relevant groupings	N/A
Population characteristics	N/A
Recruitment	N/A
Ethics oversight	N/A

Note that full information on the approval of the study protocol must also be provided in the manuscript.

Field-specific reporting

Please select the one below that is the best fit for your research. If you are not sure, read the appropriate sections before making your selection.

☒ Life sciences ☐ Behavioural & social sciences ☐ Ecological, evolutionary & environmental sciences

For a reference copy of the document with all sections, see nature.com/documents/nr-reporting-summary-flat.pdf

Life sciences study design

All studies must disclose on these points even when the disclosure is negative.

Sample size	Neural recording sample size comprised of 95 neurons for the main experiment recorded from from 2 monkeys. The sample sizes are comparable to those used by previous studies in the field.
Data exclusions	Neurons were excluded if they were not isolated across experimental conditions.
Replication	The experiments were replicated across 2 monkeys to ensure the reliability and reproducibility of the results. Each condition was repeated at least 10 motions repeats for consistency, in each monkey. Analyses on the population showed reproducibility of the results.
Randomization	Blocks of stimulation paradigms were presented in randomized order to ensure the minimization of order effects and bias
Blinding	Blinding is not relevant for our study because the recorded neural spike activity cannot be influenced by the experimenter.

Reporting for specific materials, systems and methods

We require information from authors about some types of materials, experimental systems and methods used in many studies. Here, indicate whether each material, system or method listed is relevant to your study. If you are not sure if a list item applies to your research, read the appropriate section before selecting a response.

Materials & experimental systems

n/a	Involved in the study	
☒	☐	Antibodies
☒	☐	Eukaryotic cell lines
☒	☐	Palaeontology and archaeology
☐	☒	Animals and other organisms
☒	☐	Clinical data
☒	☐	Dual use research of concern
☒	☐	Plants

Methods

n/a	Involved in the study	
☒	☐	ChIP-seq
☒	☐	Flow cytometry
☒	☐	MRI-based neuroimaging

Animals and other research organisms

Policy information about studies involving animals; ARRIVE guidelines recommended for reporting animal research, and Sex and Gender in Research

Laboratory animals	Two rhesus monkeys (Macaca mulatta) were included in the study. The animals were aged 7–13 years (mean: 10 years) and weighed between 8 and 11 kg (mean: 9.5 kg).

Wild animals	The study did not involve wild animals.
Reporting on sex	1 male and 1 female
Field-collected samples	The study did not involve samples collected in the field.
Ethics oversight	All experimental procedures were approved by the Johns Hopkins University Animal Care and Use Committee which is accredited by the Association for the Assessment and in compliance with the guidelines of the United States National Institutes of Health (PR22M342).

Note that full information on the approval of the study protocol must also be provided in the manuscript.

Plants

Seed stocks	N/A
Novel plant genotypes	N/A
Authentication	N/A
